# Differential regulation of receptivity in two uterine horns of a recipient mouse following asynchronous embryo transfer

**DOI:** 10.1038/srep15897

**Published:** 2015-11-04

**Authors:** Shi-Jie Li, Tong-Song Wang, Fu-Niu Qin, Zhu Huang, Xiao-Huan Liang, Fei Gao, Zhuo Song, Zeng-Ming Yang

**Affiliations:** 1 Colleage of Veterinary Medicine, South China Agricultural University, Guangzhou, China; 2 School of Life Science, Northeast Agricultural University, Harbin, China; 3 School of Science, Shantou University, Shantou, China; 4 School of Life Science, Xiamen University, Xiamen, China; 5 College of Life Science, Anqing Normal University, Anqing, China

## Abstract

Receptivity is a limited time in which uterine endometrium can establish a successful dialogue with blastocyst. This study was to investigate the effect of asynchronous embryo transfer on uterine receptivity in mice. Embryos under different stages were transferred into two oviduct sides of a recipient mouse on day 1 of pseudopregnancy. Our results showed the asynchronously transferred embryos can implant in all groups. Compared to zygote-transfer group, the length of implanted embryos is longer in 8-cell embryo- or blastocyst-transfer group. The levels of Snail and COX-2 immunostaining in blastocyst-transfer group are significantly stronger than that in zygote-transfer group. Embryos in blastocyst-transfer group migrate faster than that in zygote-transfer group within uterus. Blastocysts are in a state of developmental delay after they are transferred into oviducts, and they are reactivated and implanted rapidly in uterus. The developmental rate to newborn in zygote-transfer group is obviously higher than that in blastocyst-transfer group, suggesting that a delay in embryo development and implantation will lead to a decrease of litter size. These results indicated that the window of implantation is differentially regulated in two uterine horns of a recipient by embryos at different stages.

Embryo implantation is a process that active blastocysts act with the uterus in receptive state and finally establish a close connection with uterine endometrium. Uterus is only receptive for blastocysts in a short period of time, known as the window of implantation^1^. The success of implantation relies on the synchronization of endometrial receptivity and blastocyst activation^2^. Endometrial receptivity was first found in rats, and subsequently confirmed in other species^3^,^4^. In rodents and humans, endometrial receptivity only lasts for a limited time. Only in this period can the embryo make a connection with uterine endometrium. Thus it is crucial that embryos migrate successfully into receptive uterus at the right time^5^,^6^. Embryo adhesion takes place at 22:00–24:00 on day 4 of pregnancy in mice^7^ or on days 20–21 of menstrual cycle in humans^8^, when stromal vascular permeability is increased in sites of blastocyst adhesion^9^.

The implantation of the blastocyst into the maternal uterus is a crucial step for the successful establishment of mammalian pregnancy. The development of endometrial receptivity is primarily coordinated by maternal estrogen and progesterone^10^. Although many receptivity-related factors have been identified, there is no undisputed marker for human uterine receptivity. During *in vitro* fertilization, the fertilization rate is almost 100%, implantation rate remains disappointingly low. Endometrial receptivity now appears to be the bottleneck of the reproductive process^4^. However, the mechanism underlying uterine receptivity is still unknown.

In mice, receptivity is mainly controlled by maternal estrogen. Ovariectomy prior to embryo implantation will lead to delayed implantation. A single injection of estrogen can initiate embryo implantation in these ovariectomized mice. The duration of endometrial receptivity is determined by the dose of estrogen, and a high level of estrogen will shorten the duration of receptivity^5^. However, the window of implantation is also regulated by the activity of blastocysts. The window remains open for a shorter period for dormant blastocysts than for normal blastocysts^11^. After asynchronous embryos are transferred into the genital tract in a recipient mouse, embryos at a later stage were considered to stop their development before the implantation window is ready. In other words, embryos at different development stages will implant at the same time, at least within a few hours. During the subsequent development of later embryos, embryos will adjust to developmental state of recipient^12^,^13^. However, Ueda *et al.* showed that embryos at different development stages were not synchronously implanted in a recipient. Embryos at 8-cell stage will implant earlier than embryos at pronuclear stage when they are transferred into the oviducts of a recipient, suggesting that the window of implantation is differentially controlled for 8-cell and pronuclear embryos, respectively^14^. It is unknown how embryos at different stages regulate uterine gene expression in each uterine horn of one recipient. If more advanced blastocysts are transferred with zygote, it is unclear what will happen for both embryos and uterus. In mice and humans, a delay in embryo implantation will cause severe pregnancy outcome^15^,^16^,^17^. We are wondering whether the pregnancy outcome is different after zygotes or blastocysts were transferred into oviducts on day 1 of pseudopregnancy.

Zygote intrafallopian transfer is currently used to almost exclusively to patients with repeated implantation failure^18^. Furthermore, blastocyst intrafallopian transfer is a feasible option in a case of repeated difficult embryo transfer (regardless of whether the patient shows cervical adhesions or any type of genital malformations)^19^. However, how these intrafallopian transferred embryos interact with uterus is still unknown. In this study, we transferred both blastocysts and zygotes, or both 8-cell embryos and zygotes into each oviduct of a recipient to analyze how embryos locally regulate uterine receptivity. Our data suggested that the window of implantation is not only regulated by maternal hormones, but also locally controlled by implanting embryos.

## Results

### Implantation rate of embryos at different developmental stages

**Figure 1:**
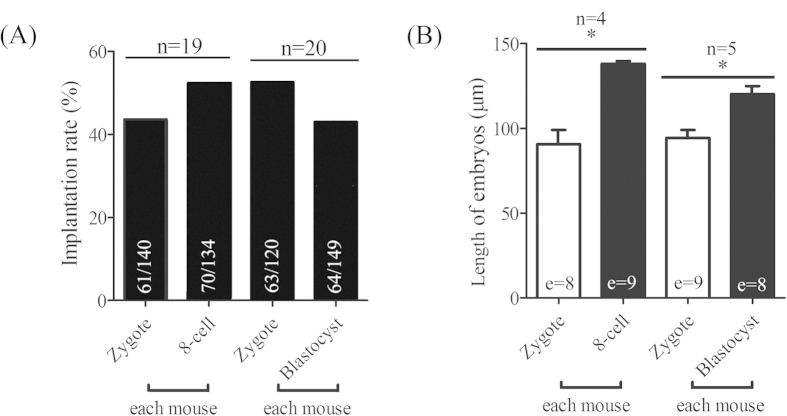
Implantation rate of embryos transferred at different development stage. (**A**) The implantation rate of transplanted zygote-8-cell group in study 1 and zygote-blastocyst group in study 2 on day 5. The number of implanted embryos (a) and the number of transferred embryos (b) are shown in each column as a/b. (**B**) The length of embryos in zygote-8-cell embryo group and zygote-blastocyst group. (e) the number of embryos used for measuring the length of implanted embryos. n, the number of recipients used.

We analyzed the implantation potential of embryos at different stages in a recipient. In study 1, zygotes and 8-cell embryos were transferred into each oviduct of a day 1 pseudopregnant recipient, respectively. The implantation rate on day 5 was 43.57% for zygote-transfer group and 52.24% for 8-cell embryo-transfer group (Fig. 1A). In study 2, zygotes and blastocysts were transferred into each oviduct of a day 1 pseudopregnant recipient. The implantation rate on day 5 was 52.5% for zygote-transfer group and 42.95% for blastocyst-transfer group (Fig. 1A). These data showed that embryos at different stages could implant after transferred into each oviduct of a recipient.

### The length of implanted embryos following nonsynchronous embryo transfer

Although embryos at different stages could implant after transferred into day 1 pseudopregnant oviducts, we found that the length of implanted embryos was different among them. In both study 1 and study 2, the implantation sites transferred with 8-cell embryos and blastocysts were bigger than that with zygotes at 8:00 on day 5. The length of implanted embryos was 90.7 ± 10.1 μm for zygotes and 138.0 ± 1.6 μm for 8-cell embryo in zygote-8-cell embryo group (Fig. 1B). In zygote-blastocyst group, the length of embryo was 94.4 ± 7.3 μm for zygotes and 120.0 ± 7.2 μm for blastocysts (Fig. 1B). These data suggested that the difference in the length of implanted embryos may come from different time of implantation.

### Nonsynchronous implantation of embryos at different stages

**Figure 2:**
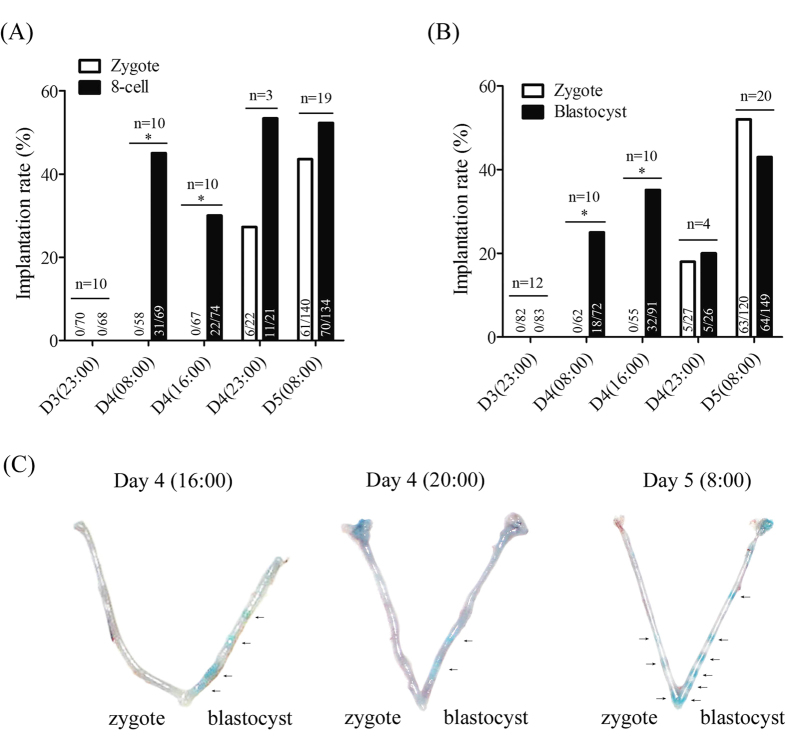
Implantation rate of embryos transferred into each mouse at different development stage. (**A**) The implantation rate following the transfer of zygotes and 8-cell embryos into the left and right oviducts of a recipient in study 1 at different time points. The number of implanted embryos (a) and the number of transferred embryos (b) are shown in each column as a/b. (**B**) The implantation rate following the transfer of zygotes and blastocysts into the left and right oviducts of a recipient in study 2 at different time points. The number of implanted embryos (a) and the number of transferred embryos (b) are shown in each column as a/b. (**C**) The representative pictures of implantation sites following the transfer of zygotes and blastocysts into the left and right oviducts of a recipient in study 2 at different time points. n, the number of recipients used. *P < 0.05; error bars, S.E.

Then we examined the implantation sites at different time points after embryo transfer. At 23:00 on day 3, implantation sites were not detected in all of the groups. At 08:00 and 16:00 on day 4, implantation sites were not detected after zygotes were transferred, but implantation rates were 44.9% at 08:00 and 29.7% at 16:00 after 8-cell embryos were transferred in zygote-8-cell embryo group (Fig. 2A). Similarly, in zygote-blastocyst group, implantation sites were not detected at 08:00 and 16:00 on day 4 after zygotes were transferred, but implantation rates were 25% at 08:00 and 35.1% at 16:00 after blastocysts were transferred (Fig. 2B). When we examined implantation sites at 23:00 on day 4 and 08:00 on day 5, respectively, implantation sites were detected in all of the groups (Fig. 2A,B). Our data indicated that blastocysts or 8-cell embryos implanted earlier than zygotes when they were transferred into each oviducts of a recipient (Fig. 2A–C).

### The expression of implantation-associated molecules

**Figure 3:**
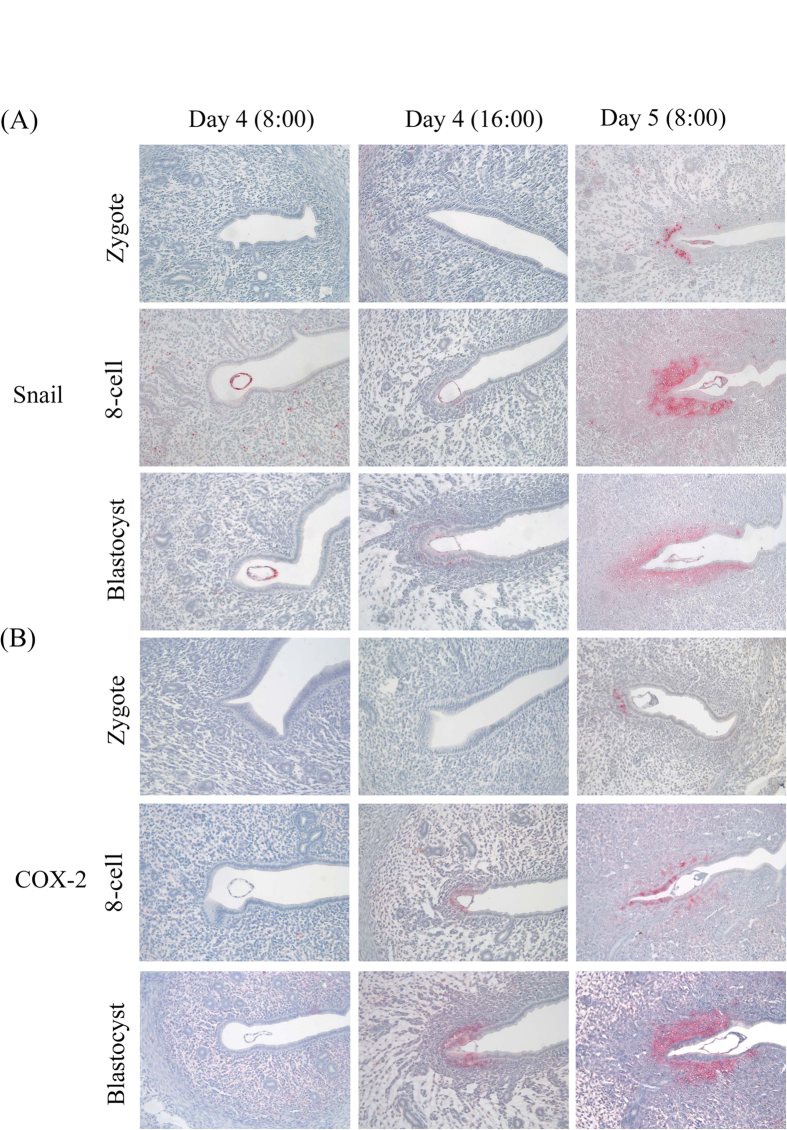
The expression of implantation-associated molecules. (**A**) Immunohistochemistry of Snail. (**B**) Immunohistochemistry of COX-2.

Because blastocysts or 8-cell embryos may implant earlier than zygotes in a recipient mouse, we wondered whether implantation-related genes (COX-2 and Snail) are differentially expressed in each uterine horns following nonsynchronous embryo transfer into a recipient. In zygote-blastocyst group, Snail was detected in the subluminal stromal cells at 16:00 on day 4 at implantation sites following blastocyst transfer, but Snail signal was not detected until 8:00 on day 5 at implantation sites following zygote transfer (Fig. 3A). The expression of COX-2 was the same situation as Snail (Fig. 3B). COX-2 immunostaining was also detected earlier in blastocyst transfer group than zygote-transfer group. These data suggested that the expression of Snail and COX-2 in each uterine horn of a mouse should be separately regulated by embryos following zygote- or blastocyst-transfer.

### Migration of embryos after asynchronous transfer

**Figure 4:**
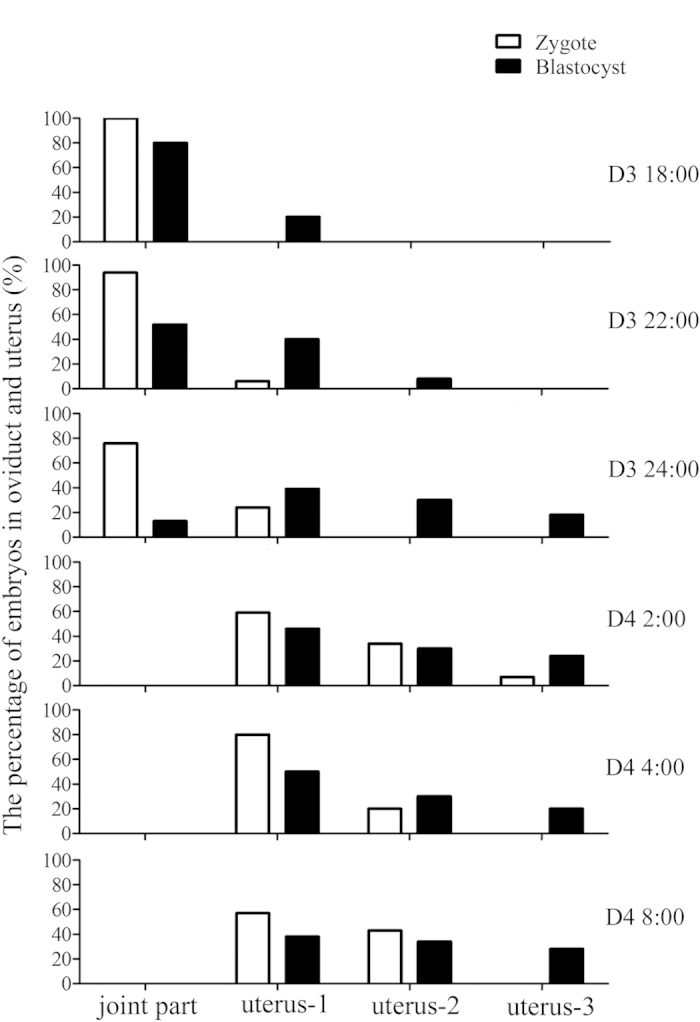
Migration of zygote and blastocyst after asynchronous transfer. The uterus was divided into three equal parts, and the uterus-1 referred to the part near oviduct.

After examined the location of embryos in oviducts and uteri at different time points, we found that the migration of embryos was different between zygote- and blastocyst-transfer groups, showing that embryos in blastocyst-transfer group migrated much faster than zygote-transfer group (Fig. 4). Our finding indicated the transplanted blastocysts could be more rapid ready for implantation.

### Proliferation of transferred blastocysts

**Figure 5:**
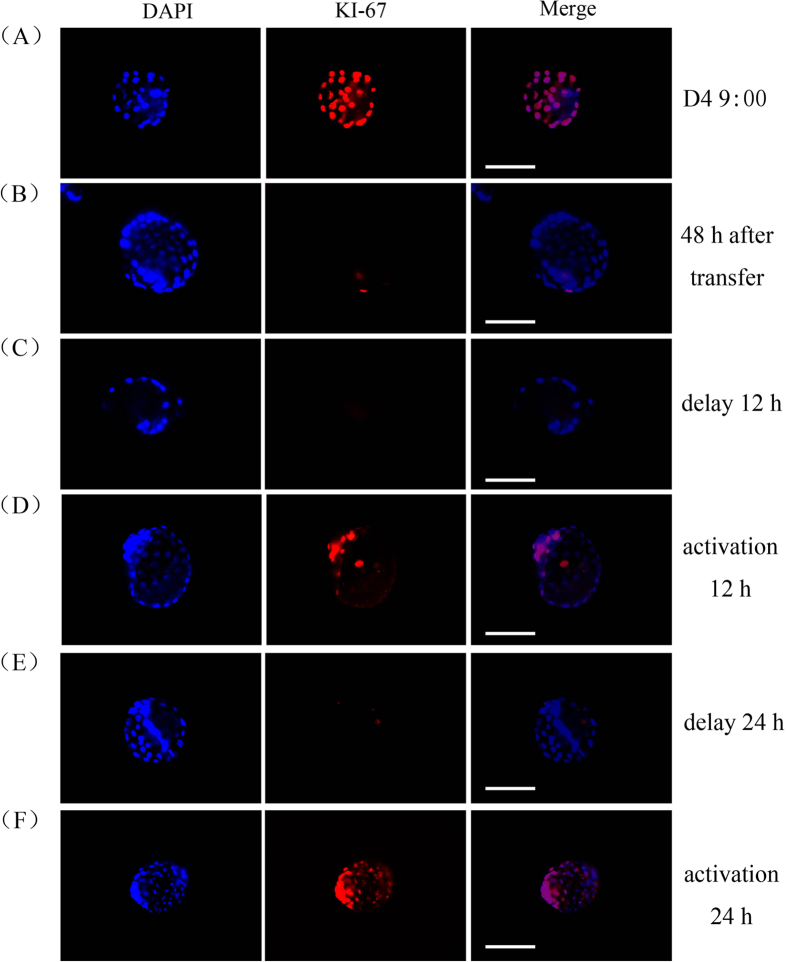
The representative Ki67 immunofluorescence of blastocysts under delayed and activated states. (**A**) Immunofluorescence of KI67 on blastocysts flushed from day 4 pregnancy uteri. (**B**) Immunofluorescence of KI67 on blastocysts which were transferred into pseudopregnancy mouse and collected on day 3. (**C**) Immunofluorescence of KI67 on blastocysts which were delayed 12 hours. (**D**) Immunofluorescence of KI67 on blastocysts which were activated 12 hours. (**E**) Immunofluorescence of KI67 on blastocysts which were delayed 24 hours. (**F**) Immunofluorescence of KI67 on blastocysts which were activated 24 hours. For the Ki67 immunofluorescence in each group, at least 5 blastocysts from each mouse were observed and 3 mice were used. A representative Ki67 immunofluorescence was shown. Scale bar, 100 μm.

Ki67 immunostaining was stronger in blastocysts flushed from normal mice on day 4 pregnancy (Fig. 5A). But after blastocysts were transferred into the oviducts on day 1 of pseudopregnancy and flushed from uterus on day 3, there was no detectable Ki67 immunostaining in these flushed blastocysts (Fig. 5B). We also examined Ki67 immunostaining in delayed and activated blastocysts for a comparison. KI67 immunostaining was detected in the activated blastocysts (Fig. 5D,F), but not in delayed blastocysts (Fig. 5C,E). Based on our Ki67 immunostaining data, we thought that the blastocysts flushed on day 3 should be in a state of developmental delay after they were transferred into oviducts.

### Litter size of nonsynchronously transferred embryos

**Figure 6:**
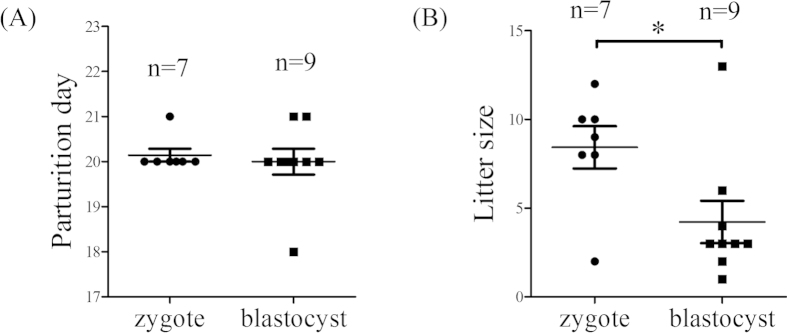
The parturition and litter size of transferred embryos at different stages. (**A**) The parturition time in zygote-transfer group and blastocyst-transfer group. (**B**) The litter size in zygote-transfer group and blastocyst-transfer group. n, the number of recipients used. *P < 0.05; error bars, S.E.

We wondered whether blastocysts or zygotes transferred into day 1 pseudopregnant oviducts could normally develop to term. We didn’t notice any differences on day 5 between zygote- and blastocyst-transfer groups. Although the time of parturition was similar between two groups (Fig. 6A), the litter size in zygote-transfer group was obviously higher than that of blastocyst-transfer group (Fig. 6B), suggesting that a delay in blastocyst-transfer group led to a decrease of litter size. Our results also indicated that the early stage of embryo was beneficial to pregnancy during embryo transfer.

## Discussion

It is generally acknowledged that the establishment of uterine receptivity is mainly regulation by steroid hormones^1^,^20^. However, the window of implantation remains open for a shorter period for dormant blastocysts than for normal blastocysts, suggesting that this window is also regulated by the activity of blastocysts^11^. Nevertheless, the whole environment in both uterine horns is regulated by the activity of blastocysts. In our study, embryos in each uterine horn implanted into uterine endometrium at a different time in a single mouse, suggesting that the microenvironment in each uterine horn should be regulated separately by embryos transferred into each corresponding oviduct, respectively. Although the duration of implantation window is determined by maternal estrogen^5^, the concentration of maternal estrogen in two uterine horns of one mouse should be the same or similar. These transferred blastocysts will induce the window of implantation earlier than zygotes although maternal estrogen concentration should be the same in one recipient. Estrogen produced by blastocysts may initiate similar attachment reaction like maternal estrogen. However, mouse blastocysts fail to produce estrogen although pig blastocysts can produce a great amount of estrogen^21^. It is possible that implantation serine proteinases produced by blastocysts may initiate the zona hatching and embryo implantation at a local environment^22^. Data from *in vitro* fertilization and embryo transfer (IVF–ET) suggest that embryo which implants first will induce the change of uterine receptivity, helping the subsequent embryo implantation when two or more blastocysts are transferred into the uterus^23^,^24^. It remains to determine how transferred blastocysts locally regulate the window of implantation.

In human beings, delayed implantation will lead to a high pregnancy loss^16^. In mice, cPLA2a deficiency causes a delay in embryo implantation, leading to small litter size and parturition^15^. LPA3 knockout mice also show delayed implantation and altered embryo spacing, resulting in significantly reduced litter size^17^. These data suggested that timing implantation is essential for pregnancy and delayed implantation will lead to bad pregnancy outcomes. Our data showed that parturition time is similar for zygotes and blastocysts. However, litter size is different between blastocyst-transferred and zygote-transferred groups. The litter size with zygote transfer is significantly higher than blastocyst transfer, suggesting the temporal dormancy of blastocysts in oviducts may produce subsequent detrimental effects on embryonic development or placental development. After blastocysts were transferred into the oviduct, these blastocysts will undergo dormancy without cell proliferation, similar to delayed blastocysts.

It was previously thought that when embryos at the early and late development stages were both transferred into the genital tract of the recipient mice, the advanced developed embryos will temporarily stop developing until uterine implantation window is ready and embryos at different developmental stages will implant at the same time^12^,^13^. Embryonic development will be retarded after embryos are transferred to ‘younger’ uteri. However, embryonic development will be accelerated when embryos are transferred to ‘older’ uteri^25^. In our study, no cell proliferation was detected in the collected blastocysts 48 h after blastocysts were transferred into oviducts on day 1 of pseudopregnancy. Blastocysts transferred into oviducts should be at a dormant state. The same situation is observed after sheep blastocysts are transferred into pseudopregnant recipient mice^26^.

COX-2 is specifically expressed in luminal epithelium and stroma around the implantation embryos^27^,^28^. COX-2 deficiency will cause the failure of embryo implantation and decidualization^29^. At 16:00 on day 4, COX-2 is detected in the luminal epithelium and subluminal stroma around implanting blastocyst in blastocyst group, but not detected at zygote group. At 08:00 on day 5, COX-2 immunostaining at implantation site is significantly stronger at blastocyst-transfer side than at zygote-transfer side. Similar expression pattern is detected for Snail immunostaining. These data indicated that uterine expression of COX-2 and Snail is locally regulated by implanting embryos in each uterine horn even if in one recipient. Although zygotes and blastocysts migrate in a similar speed in each oviduct of one recipient, blastocysts migrated faster than zygotes in uteri.

In conclusion, our data revealed that late stage embryos implant faster than early stage embryos after transfer, speed up embryo migration in the uterus, and induce the early expression of implantation-related molecules Snail and COX-2, when compared with early-stage embryos after asynchronous transfer into one recipient. Remarkably, the blastocysts were in a state of developmental delay after they were transplanted into oviducts. Not until the delayed blastocysts migrated into uterus, they were reactivated and implanted rapidly. However, it seemed that the development stage of embryos did not affect implantation rate, and early-stage of embryos were beneficial to pregnancy during embryo transfer. Our research is helpful to further understand the process of embryo implantation. For how the transferred embryos locally regulate the window of implantation requires further study to prove.

## Materials and Methods

### Animal treatments

Mature Kunming White mice (8–12 weeks old for female and 10 weeks for male) were purchased from Experimental Animal Center of Second Affiliated Hospital of Harbin Medical University (Harbin, China) and maintained on a light/dark cycle (12 h light/12 h dark) with free access to regular food and water in a controlled environment (22–24 °C and 60–70% relative humidity). All animal protocols were approved by the Animal Care and Use Committee of Northeast Agricultural University. All of the experiments were carried out in accordance with the approved guidelines by Northeast Agricultural University.

### Induction of delayed implantation

Pregnant mice were anaesthetized and ovariectomized at 08:30–09:00 on day 4 of pregnancy to induce delayed implantation. Delayed implantation was maintained by daily s.c. progesterone treatment (1 mg/mouse; Sigma) on days 5–7. To terminate delayed implantation, these mice were treated with estradiol-17β (25 ng/mouse, s.c.; Sigma) on day 7. Mice were sacrificed and uteri were collected 24 h later following estrogen treatment. Delayed implantation was confirmed by flushing blastocysts from one horn of the uterus.

### Collection of donor embryos

Female mice were mated with male mice in the afternoon. The day when vaginal plug was detected was defined as day 1 of pregnancy. Zygotes and 8-cell embryos were collected respectively at 11:00 on day 1 and 11:00 on day 3 by flushing the fallopian tube. Blastocysts were collected at 11:00 on day 4 of pregnancy by flushing uteri. These collected embryos were cultured in M2 medium under 5% CO2 incubator at 37 °C.

### Embryo transfer

Female mice were mated with vasectomized males to induce pseudopregnancy (day 1 is the day of vaginal plug). In study 1, zygotes and 8-cell embryos were transferred into left and right oviducts of a recipient on day 1 of pseudopregnancy, respectively. In study 2, zygotes and blastocysts were transferred into left and right oviducts of a recipient on day 1 of pseudopregnancy. All of embryo transfer experiments were performed at 12:00 ~ 14:00 on day 1 of pseudopregnancy..

### Embryo implantation detection and section harvest

Embryo implantation on day 5 was determined through tail-vein injection of 1% Chicago blue dye (Sigma) in saline at 8:00 ~ 9:00 on day 5 of pregnancy. Uteri were collected 5 min after injection of Chicago blue dye to visualize the implantation sites. The implantation sites were fixed by Bouin’s fixative (saturated water solution of picric acid: 37% ~ 40% formaldehyde: glacial acetic acid = 15:5:1) for 24 h, embedded in paraffin, and finally used in histological and immunohistochemical experiments.

### Embryo migration

The collected zygotes and blastocysts were stained with fluorescent dye- Cell Tracker Red CMFDA (Invitrogen, Carlsbad, CA) according to the manufacturer’s instruction, and then transferred into pseudopregnant female recipients. The migration of embryos in the fallopian tubes and uterus was examined under fluorescence microscopy and recorded the location of each embryo in uterine horns from 18:00 on day 3 to 8:00 on day 4.

### Hematoxylin-eosin staining

The 7 μm paraffin sections were cut and stained with Harris hematoxylin solution for 1 min. After differentiating sections in 1% acid alcohol for 30 sec, sections were counterstained in eosin solution for 30 sec. Sections were dehydrated and mounted with mounting medium.

### Immunohistochemistry

The paraffin sections (7 μm thick) were deparaffinized in xylene and hydrated through graded ethanol. After blocked with 10% horse serum at 37 °C for 1 h to prevent nonspecific binding, sections were incubated with the goat anti-COX-2 (1:200, #4842s, Cell Signaling Technology, Boston, MA) or goat anti-Snail antibody (1:400; sc-10432, Santa Cruz, USA) diluted in PBS for 1 h. Following three washes in PBS, sections were incubated with biotin-coupled rabbit anti-goat IgG antibody (Vector Laboratories, Burlingame, CA) for 30 min and subsequently incubated with alkaline phosphatase-coupled streptavidin (Vector Laboratories) for 30 min. The positive signal was visualized as red color by Vector Red according to the manufacturer’s protocol (Vectastain ABC-AP kit, Vector Laboratories). Endogenous alkaline phosphatase activity was inhibited with levamisole. The sections were counterstained with hematoxylin and examined under a microscopy.

### Immunofluorescence staining

Mouse blastocysts were fixed in 4% formaldehyde/PBS, and washed in 0.1% BSA/PBS. After washing in 0.1% Triton X-100/1% BSA in PBS, blastocysts were blocked with 10% horse serum for 1 h at 37 °C, and then incubated with anti-KI67 antibody (1:100, BD Pharmingen, San Diego, USA) for 1 h at 37 °C. Ki67 immunostaining was used to be a marker for cell proliferation^30^. After washing in 0.1% BSA/PBS, blastocysts were incubated with TRITC-conjugated goat anti-mouse antibody (Zhongshan Golden Bridge, Beijing, China) and counterstained with DAPI for nuclei. The fluorescent signals were examined under a fluorescence microscopy. For the Ki67 immunofluorescence in each group, at least 5 blastocysts from each mouse were observed and 3 mice were used.

### Embryo transfer and developmental rate to newborn

After zygotes or blastocysts were transplanted into the oviducts of pseudopregnancy recipient mouse on day 1 of pseudopregnancy, the litter size and time of parturition were examined.

### Statistical analysis

The length of an implanting embryo is the total thickness of sections containing the long axis of embryos. The number (n) of recipients used was shown in each figure. The number (a) of implanted embryos and the number (b) of transferred embryos were shown in each group as a/b. P value < 0.05 was considered statistically significant. All analyses were performed using the GraphPad Prism® (GraphPad Software Inc., San Diego, CA, USA) software.

## Additional Information

**How to cite this article**: Li, S.-J. *et al.* Differential regulation of receptivity in two uterine horns of a recipient mouse following asynchronous embryo transfer. *Sci. Rep.* **5**, 15897; doi: 10.1038/srep15897 (2015).
